# Insights from Modeling the 3D Structure of New Delhi Metallo-β-Lactamse and Its Binding Interactions with Antibiotic Drugs

**DOI:** 10.1371/journal.pone.0018414

**Published:** 2011-04-11

**Authors:** Jing-Fang Wang, Kuo-Chen Chou

**Affiliations:** 1 Key Laboratory of Systems Biomedicine (Ministry of Education), Shanghai Center for Systems Biomedicine, Shanghai Jiao Tong University, Shanghai, China; 2 Shanghai Center for Bioinformation and Technology, Shanghai, China; 3 Gordon Life Science Institute, San Diego, California, United States of America; University of Cape Town, South Africa

## Abstract

New Delhi metallo-beta-lactamase (NDM-1) is an enzyme that makes bacteria resistant to a broad range of beta-lactam antibiotic drugs. This is because it can inactivate most beta-lactam antibiotic drugs by hydrolyzing them. For in-depth understanding of the hydrolysis mechanism, the three-dimensional structure of NDM-1 was developed. With such a structural frame, two enzyme-ligand complexes were derived by respectively docking Imipenem and Meropenem (two typical beta-lactam antibiotic drugs) to the NDM-1 receptor. It was revealed from the NDM-1/Imipenem complex that the antibiotic drug was hydrolyzed while sitting in a binding pocket of NDM-1 formed by nine residues. And for the case of NDM-1/Meropenem complex, the antibiotic drug was hydrolyzed in a binding pocket formed by twelve residues. All these constituent residues of the two binding pockets were explicitly defined and graphically labeled. It is anticipated that the findings reported here may provide useful insights for developing new antibiotic drugs to overcome the resistance problem.

## Introduction

The rapid growth of antibiotic resistance has become a main clinical and epidemiological problem for human health [1]. In bacteria, β-lactam antibiotics are primarily hydrolyzed by β-lactamases in an acylation-deacylation-based process [2]. Thus, it is believed that β-lactamases play an important role in leading to resistance of bacteria to β-lactam antibiotics. These enzymes are capable of cleaving the amide bond of the β-lactam ring so as to inactivate the β-lactam antibiotic drugs. According to their sequence similarities, β-lactamases can be generally divided into four classes, named as A, B, C, and D. Classes A, C, and D of β-lactamases contain serine groups in their active sites, while the enzymes in class B are metalloproteins, or called “metallo-β-lactamases”, that require one or two zinc ions for their activity. Among all the β-lactamases, metallo- β-lactamases are the major culprit causing bacteria to resist antibiotics, due to the reason that they can degrade all β-lactams except monobactams and that they are special for their constant and efficient carbapenemase activity [3].

In December 2009 a novel metallo-β-lactamase was identified in a patient hospitalized in New Delhi with an infection caused by klebsiella pneumonia [4]. This β-lactamase was later detected in bacteria in India, Pakistan, United Kingdom, United States, and Canada. The New Delhi metallo-β-lactamase (NDM-1) has the ability to make bacteria resistant to a wide range of β-lactam antibiotics, including the carbapenem family antibiotics that are a mainstay for the treatment of antibiotic-resistant bacterial infections [5], [6]. According to the report of the United Kingdom's Health Protection Agency, most isolates with NDM-1 are resistant to all standard intravenous antibiotics for the treatment of severe infections. The most common bacteria that make this enzyme are Gram negative such as Escherichia coli and Klebsiella pneumonia, but the gene for NDM-1 can spread from one strain of bacteria to another by horizontal gene transfer.

To reveal the resistance mechanism of bacteria to β-lactam antibiotics due to the existence of NDM-1, an indispensable knowledge is of the 3D (three-dimensional) structure of NDM-1. Since so far no 3D structure whatsoever has been determined by experiments for NDM-1, we have to resort to the approach of structural bioinformatics [7]. Recently, growing evidences have indicated that various tools in structural bioinformatics, such as homology modeling [8], [9], [10], [11], [12], [13], [14], [15], [16], molecular docking [17], [18], [19], [20], [21], [22], as well as molecular dynamics simulations [23], [24], [25], [26], [27], [28], [29], [30], [31], can timely provide very useful information and insights for biomedical science and drug development and hence are quite rewarding [14], [22], [29], [32], [33], [34], [35], [36], [37], [38], [39]. In view of this, the present study was initiated in an attempt to develop a homology model for NDM-1, based on which the molecular docking operations and molecular dynamics simulations were performed in hopes that the information thus obtained may provide useful insights or clues for designing new drugs to overcome the antibiotic resistance problem.

## Materials and Methods

The entire sequence of NDM-1, which contains 158 amino acids, was taken from NCBI Protein database with an accession of AB571289. According to the score of BLAST search, the crystal structure of VIM-2, a Zn-β-lactamase from Pseudomonas aeruginosa [40], was selected as a structural template to perform homology modeling to develop the 3D structure of NDM-1. The PDB code of the crystal structure is 1ko3, which was released in 2008 with a resolution of 2.20 Å [40]. The entire sequence of 1ko3 contains 230 amino acids. The sequence alignment between NDM-1 and 1ko3 was performed by the Molecular Operating Environment (MOE), and the alignment result thus obtained indicates that the two proteins have a sequence identity of 43%.

Meanwhile, using the web-server EzyPred [41] at http://www.csbio.sjtu.edu.cn/bioinf/EzyPred/ and the protein sequence information, it was identified that NDM-1 is a member of hydrolases enzyme family (acting on carbon-nitrogen bonds other than peptide bonds), and so is 1ko3. Since both NDM-1 and 1ko3 belong to a same enzyme family with the same action mechanism, and their sequence identity is higher than 40%, it is quite reasonable to use the crystal structure of 1ko3 [40] as a template to develop the 3D structure of NDM-1 via homology modeling.

**Figure 1:**
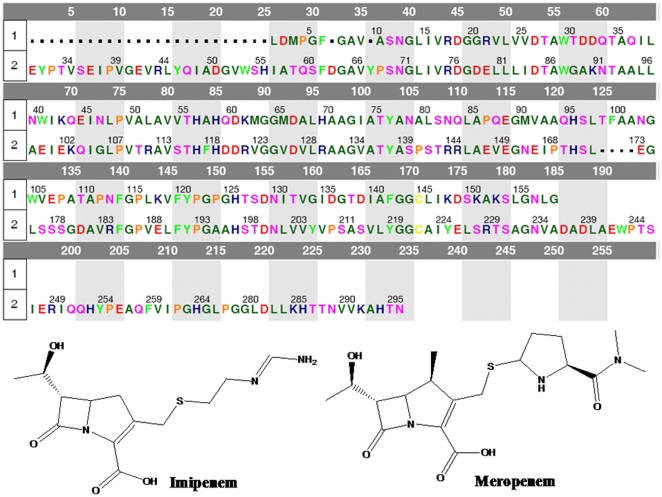
The sequence alignment of NDM-1 (chain-1) with 1ko3.pdb (chain-2); shown in the lower panel are the chemical structures of Imipenem and Meropenem. The amino acids in this figure are colored according to the following four types: (1) acidic, red; (2) basic, dark blue; (3) hydrophobic, green; (4) hydrophilic, light blue. The sequence identity of the two proteins is about 43%.

**Figure 2:**
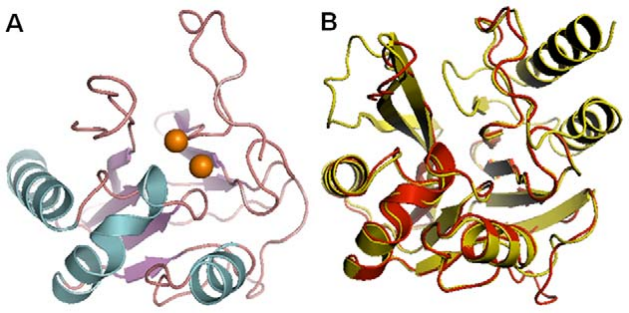
Illustration to show (A) the computed 3D structure of NDM-1 that contains three helices and 7 β-strands, and (B) a superimposition of the homology modeled NDM-1 structure (red) with its template 1ko3 (yellow).

Based on the sequence alignment (Fig. 1) as well as the atomic coordinates of 1ko3, the 3D structure of NDM-1 was derived by using the I-TASSER algorithm, an extension of the previous TASSER (Threading/Assembly/Refinement) method [42], [43], [44]. The homolog-modeled 3D structure was subject to a short-time molecular dynamics simulation (∼3 ns) for further refinement. The final 3D structure thus obtained for NDM-1 is shown in Fig. 2. Meanwhile, some assessments by the Swiss Model Server indicated that the computed structure is quite reasonable and creditable.

**Figure 3:**
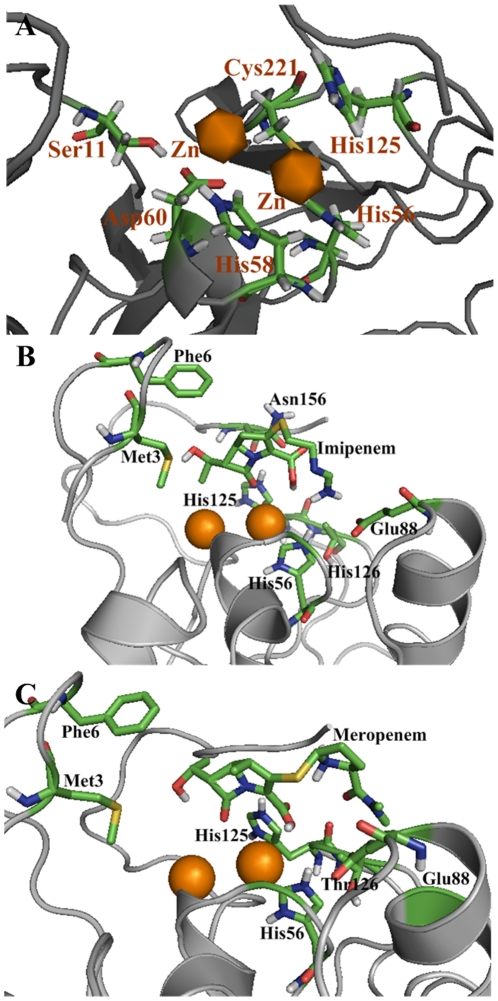
A close view to show (A) the zinc coordinates, (B) binding pocket of NDM-1 for the antibiotic drug Imipenem, and (C) binding pocket of NDM-1 for the antibiotic drug Meropenem. The binding pocket is formed by those residues that have at least one heavy atom with a distance within 5 Å [51] to Imipenem/Meropenem.

**Figure 4:**
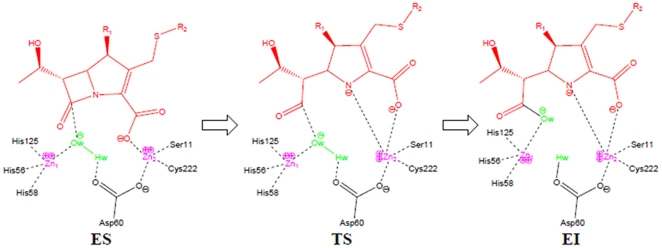
The proposed mechanism for the initial ring-opening step in the antibiotic catalysis of NDM-1, where ES, TS, and EI represent enzyme-substrate, transition state, and enzyme intermediate, respectively.

Subsequently, molecular docking operations were carried out with Monte Carlo simulated annealing [45] to get the favorable binding interaction modes for NDM-1 respectively with Imipenem and Meropenem, two typical β-lactam antibiotic drugs. It was reported recently [6] that NDM-1 showed a comparatively high resistance to Imipenem and Meropenem. The binding pocket was identified using Q-SiteFinder [46]. Because binding of inhibitors in the active site may induce a conformational change to closing some flaps [47], we adopted a flexible docking procedure to construct the binding modes of NDM-1 with Imipenem and Meropenem. Before the docking procedure, we extracted 1000 conformations of NDM-1 from the aforementioned 3-ns molecular dynamics simulation. The ligand (Imipenem or Meropenem) was then docked to all of these conformations to search for a favorable binding mode. The docking program [48] used in this study would automatically generate a diversified set of configurations by randomly changing the ligand's coordinates. When a new configuration of the ligand was generated, the search for the favorable binding was operated within a specified 3D box by the simulated annealing to optimize the purely spatial contacts as well as electrostatic interactions. Finally, the favorable binding mode thus obtained was further optimized by a short time molecular dynamics simulation (∼5 ns). The binding energy was calculated using a scoring function London dG [49], [50],[51]. In all our calculations, the Merck force field (FFMM94) parameters were adopted. The most favorable binding interactions thus obtained for NDM-1 with Imipenem and Meropenem are given in Fig. 3 and Fig. 4, respectively.

## Results and Discussion

### Homology-Modeled 3D Structure of NDM-1

A total of 100 homology-modeled structures for NDM-1 were derived with each having a C-score. The C-score is a “confidence score” for estimating the quality of a computed model: a high C-score signifies a model with a high confidence and vice-versa. Shown in Fig. 2A is the structure with the highest C-score that was used as a receptor for further docking studies. Meanwhile, we employed PROCHECK [52] to estimate the quality of our models. It was indicated by PROCHECK that there are 93.1% residues located in the “core” and “allowed” regions, 4.6% in the “general” region and only 2.3% in the “disallowed” regions (Fig. S1). In the computational structure, all the bond lengths for the main-chain residues and 91.9% bond angles for the main-chain residues are within the allowed limits. To further examine our computational model of NDM-1, we also used the tool of QMEAN, which is a scoring function of a linear combination of 6 structural descriptors [53]. The QMEAN score ranges between 0 and 1 with higher value to reflect a better quality of the model. The QMEAN score for the current computational model is 0.67, and the density plot of this QMEAN score is shown in Fig. S2. To evaluate the absolute model quality, we also calculated a Z-score of the computational model in comparison with the scores of the reference X-ray structures of similar size from the Protein Data Bank (Fig. S3). For each of the QMEAN components, a Z-score was computed in comparison with the average X-ray structures (the average value is 0, as shown in Fig. S4). Based on these analyses, we further examined the per-residue error (Fig. S5), visualized using a color gradient from blue (reliable regions with estimated error below 1 Å) to red (potentially unreliable regions with estimated error above 3.5 Å). We found that the residues in the potentially unreliable regions were mainly located on the loop regions in the computational model, indicating that further molecular dynamics optimizations are needed, as described below.

However, after a 3-ns molecular dynamics simulation, all the residues in our model were in the “core” and “allowed” regions. It can be seen from Fig. 2A that, like most of the other metallo-β-lactamases, the NDM-1 protein belongs to the α/β structural class [54], with 3 helices and 7 β-strands. The 3 helices were exposed to the solvent. The N-terminal and C-terminal regions of NDM-1 can be superposed by a 180 degrees rotation around a central axis, indicating that the entire structure may have arisen from the duplication of a gene. It was found after superposing NDM-1 to its template structure 1ko3.pdb that the two structures share a quite similar backbone (Fig. 2B) owe to their high sequence identity percentage. However, some α-helices and β-strands in 1ko3.pdb are not seen in the NDM-1 model because 1ko3.pdb contains 5 helices and 11 β-strands. This is due to the sequence deletion of NDM-1 in its C-terminal and N-terminal regions. It is instructive to note that such sequence deletion might make the β-lactam antibiotic drugs easier to access the binding pocket of NDM-1.

Similar with other metallo-β-lactamases, the active site of NDM-1 presents two metal ion binding sites: His site and Cys site. Thus, two zinc ions can be detected in our homology model at both the His and Cys sites with a distance of 4.20 Å apart. According to some experimental data, the one located in the His site has a higher occupancy (1.3 fold) than that in the Cys site (Fig. 3A). The zinc ion in the His site possesses a tetrahedral coordination sphere and is coordinated by His56, His58, and His125. The other zinc ion in the Cys site has a trigonal-pyramidal coordination sphere that involves Ser11, Asp60, and Cys144.

### Binding Modes of Inactivating Antibiotic Drugs

Since metallo-β-lactamases can inactivate the β-lactam antibiotic drugs by cleaving the amide (carbon-nitrogen) bond of the β-lactam ring [2], it can provide us very useful insights about the essence of the drug resistance problem by analyzing the binding interaction modes obtained by docking Imipenem and Meropenem to NDM-1 receptor, respectively.

Shown in Fig. 3B is a close view of the binding interactions obtained by docking the antibiotic drug Imipenem to the receptor NDM-1. Its binding pocket is formed by nine residues that have at least one heavy atom with a distance within 5 Å [55] to Imipenem, as labeled in Fig. 3B. Interestingly, the constituent residues thus defined for the binding pocket are quite consistent those identified by the online web-server tool Q-SiteFinder for forming the extended cleft active site of NDM-1. In the NDM-1/Imipenem complex, Met3 and Phe6 are located on the brink of the active site, providing some van der Waals interactions to the drug molecule. It can be seen from Fig. 3B that, in addition to the van der Waals interaction of the Imipenem drug with the surrounding binding pocket residues, there are remarkable hydrogen bonds that have tightly tethered the drug to Glu88 and Thr126 of the receptor, making NDM-1 able to recognize the antibiotic drug followed by cleaving the amide bond of its β-lactam ring so as to inactivate Imipenem. The detailed information for these hydrogen bonds is listed in Table 1.

**Table 1:** Detailed information for the H bonding formed by NDM-1 and antibiotic drugs.

H – donor	H – receptor	Bond distance (Å)	Lifetime (%)
Glu88 (O_β_)	Imipenem (NH_2_)	2.89	43.8
Glu88 (O_β_)	Meropenem (NH_2_)	3.01	39.1
Thr126 (O_α_)	Imipenem (NH_2_)	3.04	19.8
Thr126 (O_α_)	Meropenem (NH_2_)	2.78	45.4

For the case of Meropenem, it can interact with almost the same residues as Imipenem. As can be seen from Fig. 3C, the binding pocket for Meropenem involves six residues, of which Glu88 and Thr126 form three hydrogen bonds with the antibiotic drug. Since there is one more five-member ring in Meropenem (Fig. 3C), some additional hydrogen bonds are needed to stabilize the ligand during the process of cleaving its amide bond and inactivate the antibiotic drug. Based on these structural findings and previous theoretical studies [56], [57], [58], we proposed a catalytic mechanism for NDM-1, as illustrated in Fig. 4. In our model, the metal-binding Asp60 acts as the general base that activates the water nucleophile, while the protonation of Asp60 results in the cleavage of its bond to the metal ion.

## Supporting Information

Figure S1 Ramachandran plot for the computational model of NDM-1 by PROCHECK.
(TIF)

Figure S2 The density plot of the QMEAN score for the computational model of NDM-1.
(TIF)

Figure S3 Estimated absolute model quality by the comparison of the QMEAN scores with the reference x-ray structures in the Protein Data Bank.
(TIF)

Figure S4 The QMEAN score components calculated based on the Z-score of each component in comparison with the average x-ray structures.
(TIF)

Figure S5 Per-residue error visualized by using a color gradient from blue (reliable region, estimated error below 1 Å) to red (potentially unreliable regions, estimated error above 3.5 Å).
(TIF)
