# Supplementary figures and images for: Insights from Modeling the 3D Structure of New Delhi Metallo-β-Lactamse and Its Binding Interactions with Antibiotic Drugs
Source: PLoS One. 2011 Apr 11;6(4):e18414. doi: 10.1371/journal.pone.0018414 (PMC3073942; doi:10.1371/journal.pone.0018414)

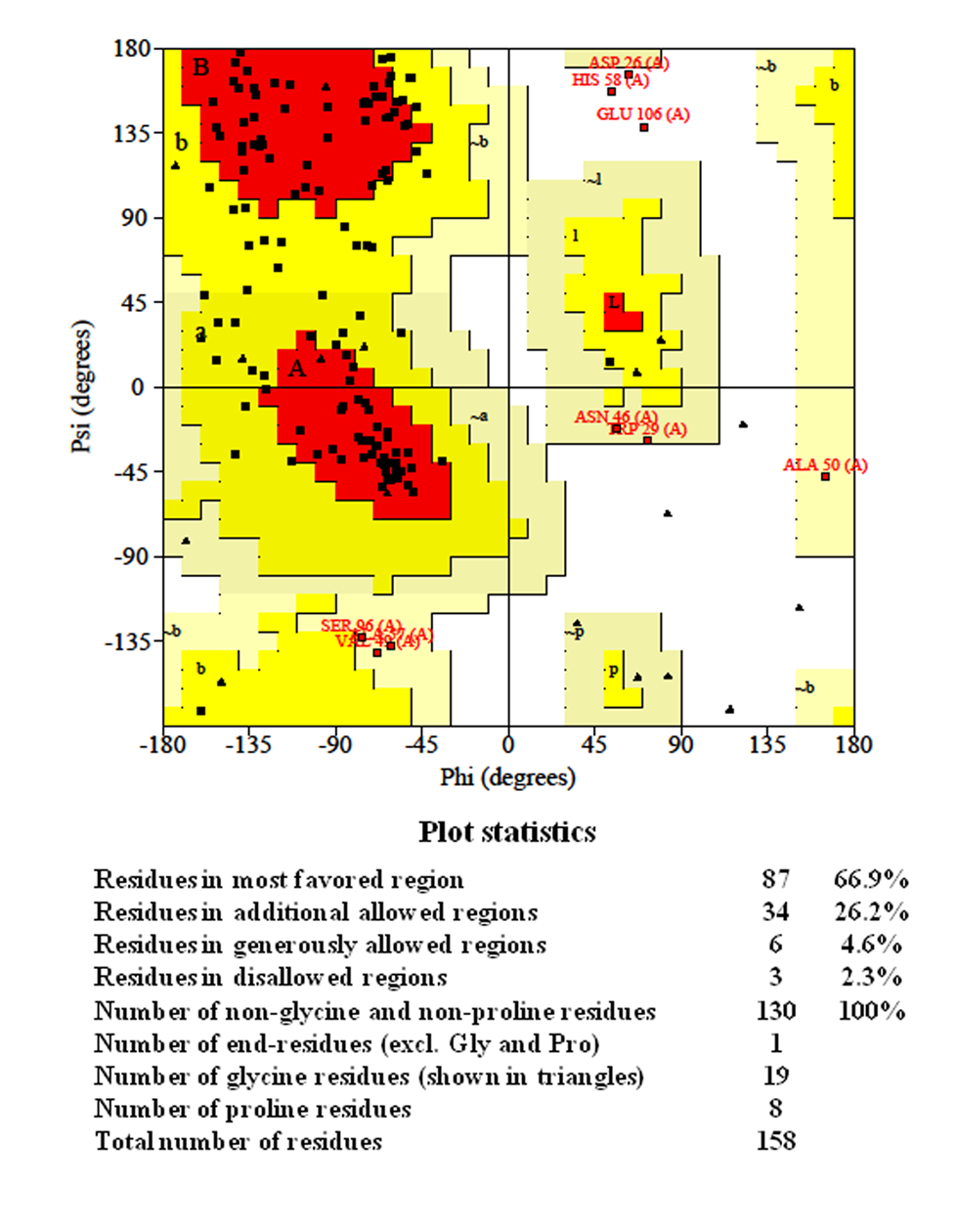

Supplement: Figure S1 — Ramachandran plot for the computational model of NDM-1 by PROCHECK. (TIF) [file pone.0018414.s001.tif]

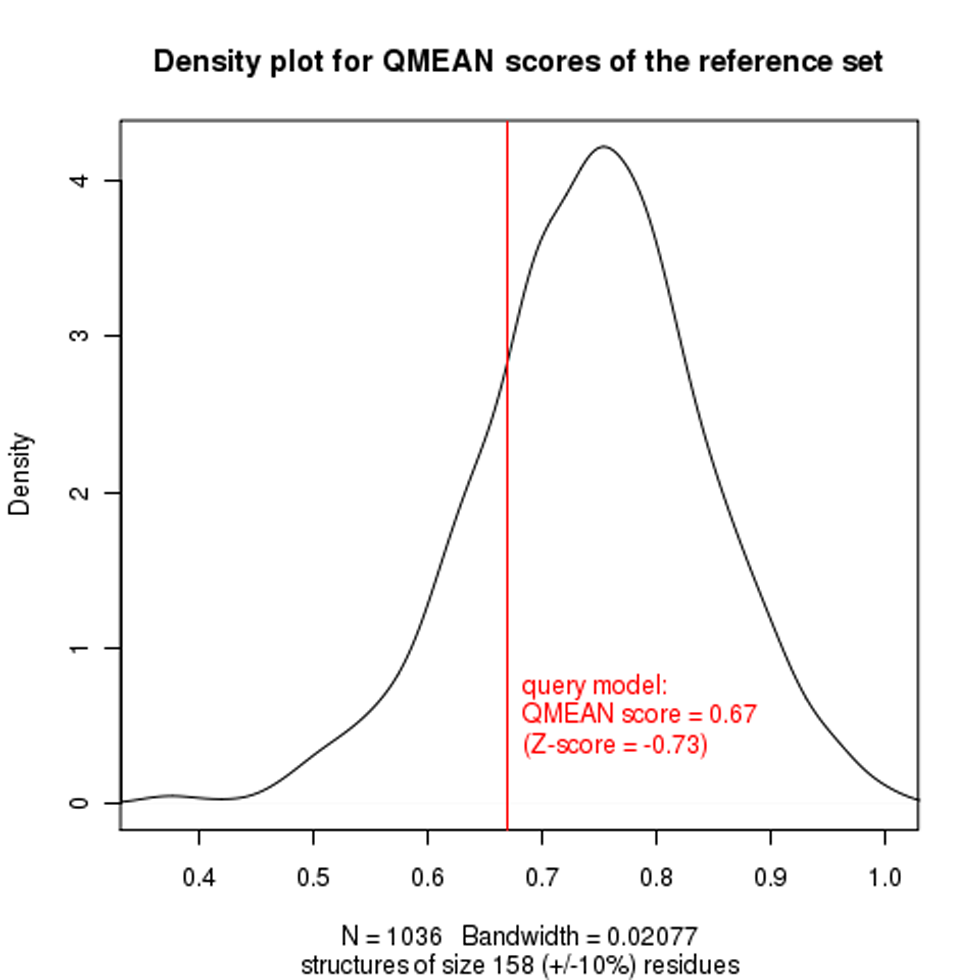

Supplement: Figure S2 — The density plot of the QMEAN score for the computational model of NDM-1. (TIF) [file pone.0018414.s002.tif]

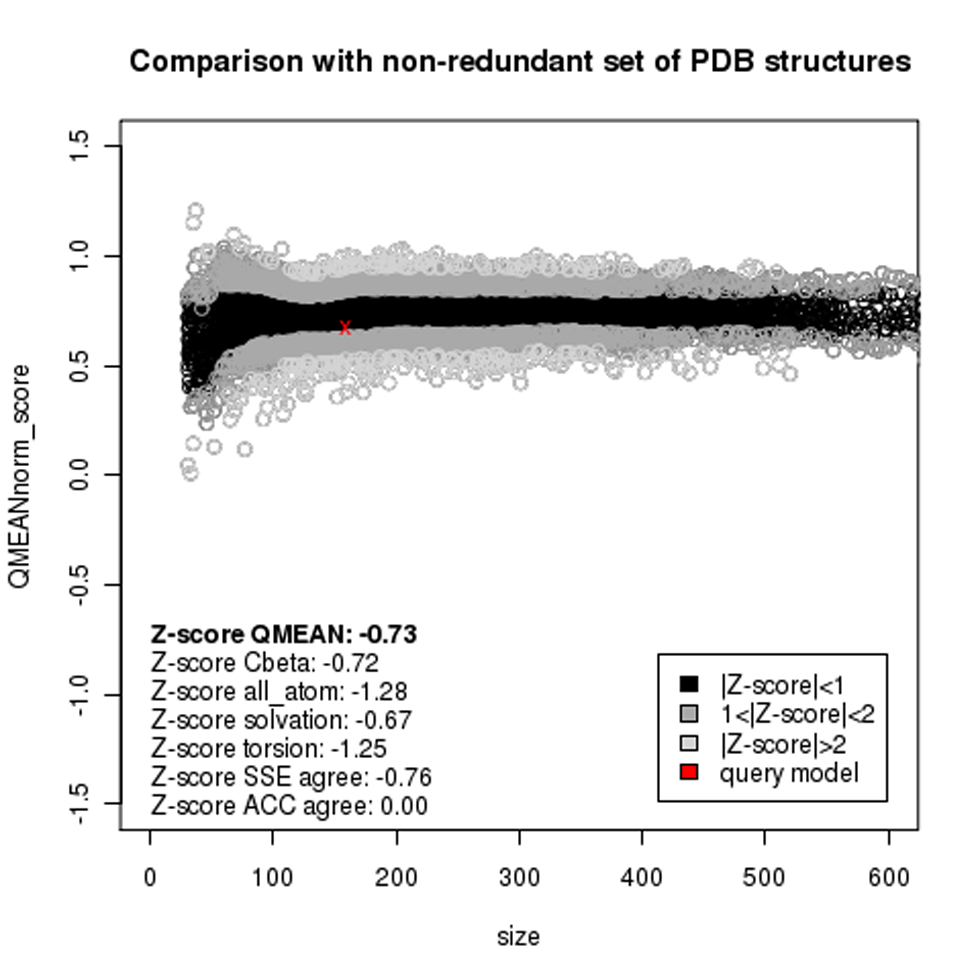

Supplement: Figure S3 — Estimated absolute model quality by the comparison of the QMEAN scores with the reference x-ray structures in the Protein Data Bank. (TIF) [file pone.0018414.s003.tif]

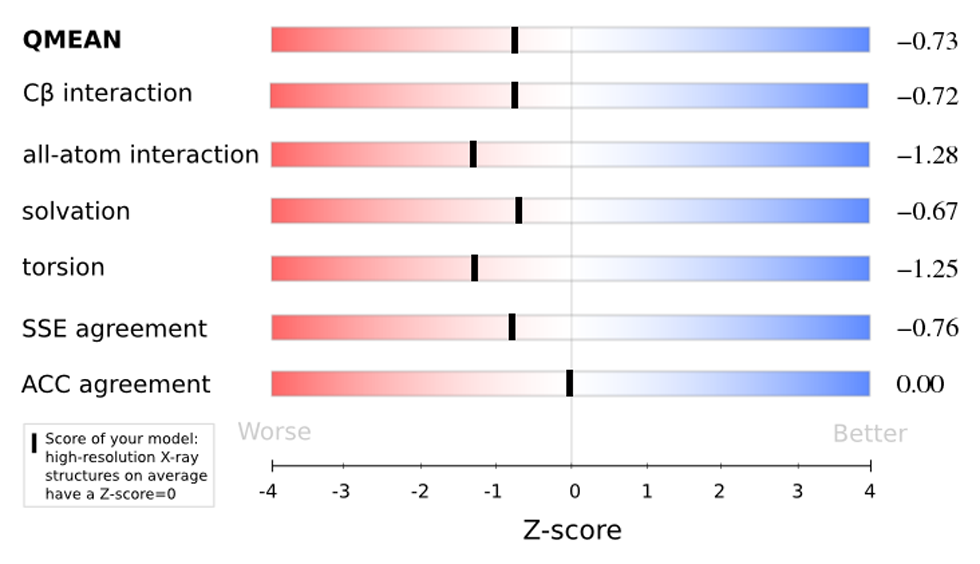

Supplement: Figure S4 — The QMEAN score components calculated based on the Z-score of each component in comparison with the average x-ray structures. (TIF) [file pone.0018414.s004.tif]

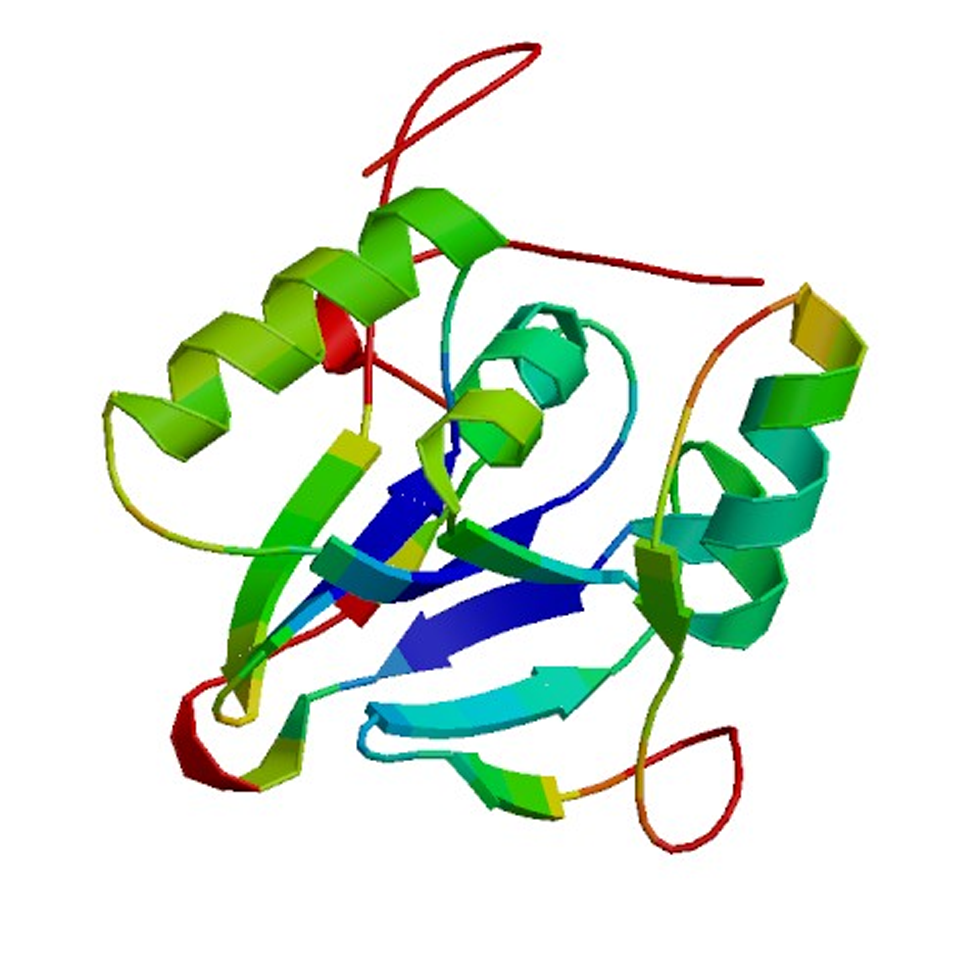

Supplement: Figure S5 — Per-residue error visualized by using a color gradient from blue (reliable region, estimated error below 1 Å) to red (potentially unreliable regions, estimated error above 3.5 Å). (TIF) [file pone.0018414.s005.tif]
